# Persistent Low-Level hCG After Gestational Trophoblastic Neoplasia Remission: Treatment-Free Probability Stratified by Early hCG Course

**DOI:** 10.3390/cancers18152434

**Published:** 2026-07-29

**Authors:** Mingliang Ji, Jun Zhao, Liangyu Xia, Xirun Wan, Fengzhi Feng, Junjun Yang, Fang Jiang, Yang Xiang

**Affiliations:** 1 National Clinical Research Center for Women’s Health and Obstetric and Gynecologic Diseases, Department of Obstetrics and Gynecology, Peking Union Medical College Hospital, Chinese Academy of Medical Sciences & Peking Union Medical College, No.1 Shuaifuyuan, Dongcheng District, Beijing 100730, China; jimingliang@pumch.cn (M.J.); wanxr@pumch.cn (X.W.); fengfzh@pumch.cn (F.F.); yangjunjun@pumch.cn (J.Y.); jiangfang@pumch.cn (F.J.); 2 Department of Laboratory Medicine, Peking Union Medical College Hospital, Chinese Academy of Medical Sciences & Peking Union Medical College, No.1 Shuaifuyuan, Dongcheng District, Beijing 100730, China; xialy@pumch.cn

**Keywords:** gestational trophoblastic neoplasia, human chorionic gonadotropin, recurrence, treatment-free probability, landmark analysis

## Abstract

**Background/Objectives**: To determine treatment-free probability after persistent low-level human chorionic gonadotropin (hCG) elevation following complete remission from gestational trophoblastic neoplasia (GTN) and examine its association with the early hCG course. **Methods**: This single-center retrospective study included patients treated in 2015–2022 for gestational choriocarcinoma or invasive mole who developed ≥3 post-remission hCG results >5 and ≤1000 U/L over ≥14 days. The primary endpoint was retreatment for GTN recurrence. Kaplan–Meier analysis estimated treatment-free probability from the first low-level hCG elevation. Exploratory landmark analyses at 60, 90, and 180 days stratified patients still under observation by pre-landmark peak hCG (≤20 versus >20 U/L). **Results**: Forty-six patients contributed 595 hCG measurements. Thirty-five developed recurrence requiring retreatment; 11 remained recurrence-free. All patients whose first low-level hCG exceeded 20 U/L (*n* = 11) developed recurrence. Treatment-free probability was 30.4% at 1 year and 24.2% at 2 years. At the 60-, 90-, and 180-day landmarks, 365-day treatment-free probability was higher with pre-landmark peak hCG ≤ 20 U/L than >20 U/L: 68.4% versus 9.1%, 70.6% versus 22.2%, and 92.3% versus 14.3%, respectively. All 35 retreated patients achieved hCG ≤ 5 U/L after treatment. **Conclusions**: Most patients with persistent low-level hCG after remission from GTN developed recurrence. For patients still under observation at 60, 90, or 180 days, a peak hCG of 20 U/L or lower before the landmark was associated with higher treatment-free probability over the following year. Continued surveillance after clinical and imaging assessment is reasonable in this subgroup.

## Simple Summary

After treatment for gestational trophoblastic neoplasia, some patients develop persistent low-level serum hCG elevation during surveillance. This may represent recurrence, but it may also remain stable or resolve without further treatment. In this single-center retrospective cohort of 46 patients, most eventually developed recurrence and received anti-GTN retreatment, while some remained treatment-free for months or years. Patients whose hCG remained at or below 20 U/L by 60, 90, or 180 days of observation had higher treatment-free probability during the following year than those whose hCG rose above 20 U/L. All retreated patients subsequently achieved hCG levels of 5 U/L or lower. The early hCG course may help distinguish patients who need retreatment from those who can continue close surveillance after clinical and imaging assessment.

## 1. Introduction

Persistent low-level human chorionic gonadotropin (hCG) elevation during surveillance after gestational trophoblastic neoplasia (GTN) treatment is a recognized but poorly defined clinical scenario. The finding may reflect early recurrence requiring treatment, but non-progressive explanations include assay interference, pituitary hCG in postmenopausal women, quiescent gestational trophoblastic disease (GTD), and transient benign hCG elevations following chemotherapy. International guidelines recommend structured evaluation including repeat measurement, alternative assay confirmation, assessment of pituitary and renal contributions, and appropriate imaging before any decision about further treatment [1,2].

Despite this guidance, the subsequent course of persistent low-level hCG after GTN remission remains poorly characterized. A prior retrospective series of 47 patients with persistent low-level hCG described varied outcomes but included placental-site trophoblastic tumor and epithelioid trophoblastic tumor, entities that may present with low hCG and require different management [3]. Data are therefore limited for patients who have achieved complete remission after treatment for gestational choriocarcinoma or invasive mole and then develop persistent low-level hCG during surveillance.

We studied patients who developed persistent low-level hCG elevation after complete remission from prior GTN treatment. The primary objective was to determine treatment-free probability and to examine whether the highest hCG value before fixed time landmarks was associated with subsequent retreatment.

## 2. Materials and Methods

### 2.1. Study Design and Setting

This was a single-center retrospective cohort study conducted at Peking Union Medical College Hospital. Eligible records were identified from January 2015 to December 2022. Follow-up extended to the last available outpatient record or hCG measurement; the latest individual follow-up date in the cohort was in February 2025. The study was approved by the institutional Ethics Committee (Approval No: I-23PJ2049), with a waiver of informed consent.

Serum hCG was measured in the clinical laboratory using the Access Total beta-hCG assay on the Beckman Coulter UniCel DxI 800 platform (Beckman Coulter, Brea, CA, USA). The clinical reporting threshold for the non-pregnant reference range is ≤5 U/L. Throughout this article, hCG refers to serum beta-hCG reported in U/L.

### 2.2. Patient Selection

Patients were included if they met all of the following: (1) prior GTN diagnosed as gestational choriocarcinoma or invasive mole; (2) achievement of complete remission after prior GTN treatment and entry into surveillance before the low-level hCG elevation; (3) at least three post-treatment hCG results >5 and ≤1000 U/L; (4) at least 14 days between the first and last such abnormal results. After inclusion, subsequent hCG values could fall below 5 U/L or exceed 1000 U/L.

**Figure 1:**
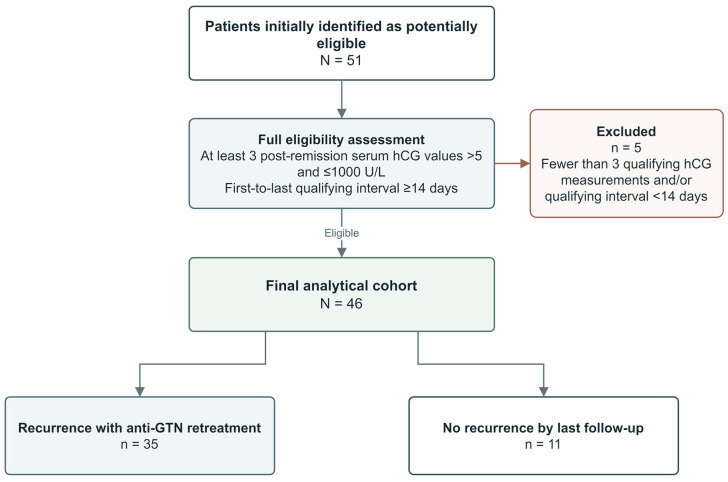
Patient selection flow diagram. The diagram shows the potentially eligible patients, exclusions with reasons, and the final cohort by recurrence status. hCG denotes human chorionic gonadotropin; GTN, gestational trophoblastic neoplasia.

Patients were excluded if the final diagnosis was placental site trophoblastic tumor, epithelioid trophoblastic tumor, or any condition other than GTN diagnosed as gestational choriocarcinoma or invasive mole [1]. Patients with insufficient follow-up data to determine whether retreatment was initiated were also excluded. The selection process is summarized in Figure 1.

### 2.3. Definitions

The date of the first low-level hCG elevation was the date of the first hCG result entering the >5 and ≤1000 U/L range during the post-remission episode. The International Federation of Gynecology and Obstetrics (FIGO) stage, FIGO score, and a background of drug-resistant or relapsed GTN were abstracted from the preceding treatment course. Menopausal status at the time of low-level hCG elevation was recorded as documented and included both natural and surgical menopause.

Recurrence was determined by the clinical team using the hCG course, imaging findings, prior disease history, symptoms when present, and clinical assessment. The event was initiation of anti-GTN retreatment for recurrence. Anti-GTN treatment included systemic chemotherapy, immunotherapy, local treatment, or their combinations within the same subsequent treatment course. Patients without recurrence by the last available record were censored at the last available follow-up or hCG record date.

Treatment-free interval was defined as the time from the first low-level hCG elevation to retreatment. Post-treatment hCG normalization was defined as at least one hCG value ≤ 5 U/L after retreatment. Post-treatment hCG measurements were counted from the end of the preceding GTN treatment to the event or censoring date and separately from the first low-level hCG elevation to the same endpoint. Clinical follow-up was measured from the first low-level hCG elevation to the last available clinical record.

### 2.4. Statistical Analysis

Continuous variables are reported as median (interquartile range [IQR]). Categorical variables are reported as counts and percentages. Baseline and early characteristics were compared by recurrence status using Fisher’s exact test for categorical variables and the Wilcoxon rank-sum test for continuous variables.

Treatment-free probability with 95% confidence intervals (CIs) was estimated by the Kaplan–Meier method at fixed time points (30, 60, 90, 180, 365, and 730 days from the first low-level hCG elevation). CIs were computed using the log–log transformation. Landmark analyses were prespecified at 60, 90, and 180 days [4,5,6]. Each analysis included patients who reached that landmark without prior retreatment. The landmark date became the new time zero, so the estimate reflected the chance of remaining treatment-free after the patient had already been observed to the selected time point. Patients were stratified by the highest hCG value recorded from the first low-level hCG elevation to the landmark as ≤20 versus >20 U/L. The 20 U/L value was derived post hoc from this cohort for exploratory stratification. The primary endpoint was 365-day treatment-free probability after the landmark, and log-rank tests were applied within this 365-day window.

To reduce small-sample bias in the landmark datasets, the association between pre-landmark peak hCG and subsequent retreatment was examined with Firth penalized Cox regression using the same 365-day post-landmark follow-up [7,8]. At the 60- and 90-day landmarks, a univariable model was fitted, followed by four models that each added one covariate: FIGO score, log10-transformed first low-level hCG, menopausal status, or prior diagnosis. Continuous first low-level hCG was interpreted per 10-fold increase. At the 180-day landmark, only a univariable model was fitted. Hazard ratios (HRs) are reported with profile-likelihood 95% CIs and likelihood-ratio *p* values.

For a sensitivity analysis, recurrence events were counted only for the 30 patients with suspicious imaging findings documented during the recurrence episode. The original treatment-decision dates were retained as event or censoring times, and the landmark Kaplan–Meier, log-rank, and univariable Firth Cox analyses were repeated. Analyses were performed in R version 4.5.2; Firth models were fitted with the coxphf package (version 1.13.4).

## 3. Results

### 3.1. Cohort Characteristics

Of 51 potentially eligible patients, five were excluded because they did not meet the complete serial low-level hCG requirement of at least three results >5 and ≤1000 U/L spanning at least 14 days. The final cohort comprised 46 patients (Figure 1). Thirty-five patients developed recurrence and received retreatment; 11 had no recurrence by the last available follow-up.

Across the post-treatment observation period, the 46 patients contributed 595 hCG measurements, with a median of 10.0 per patient (range, 5–47). When the observation window was defined from the date of the first low-level hCG elevation to the event or censoring date, 423 measurements were available, with a median of 7.5 per patient (range, 3–28).

The preceding GTN had been diagnosed as gestational choriocarcinoma in 35 patients (76.1%) and invasive mole in 11 (23.9%). Thirty-three patients (71.7%) had FIGO stage III disease and five (10.9%) had stage IV disease. Thirty patients (65.2%) had a FIGO score of 7 or higher. A background of drug-resistant or relapsed GTN was documented in 33 patients (71.7%).

**Figure 2:**
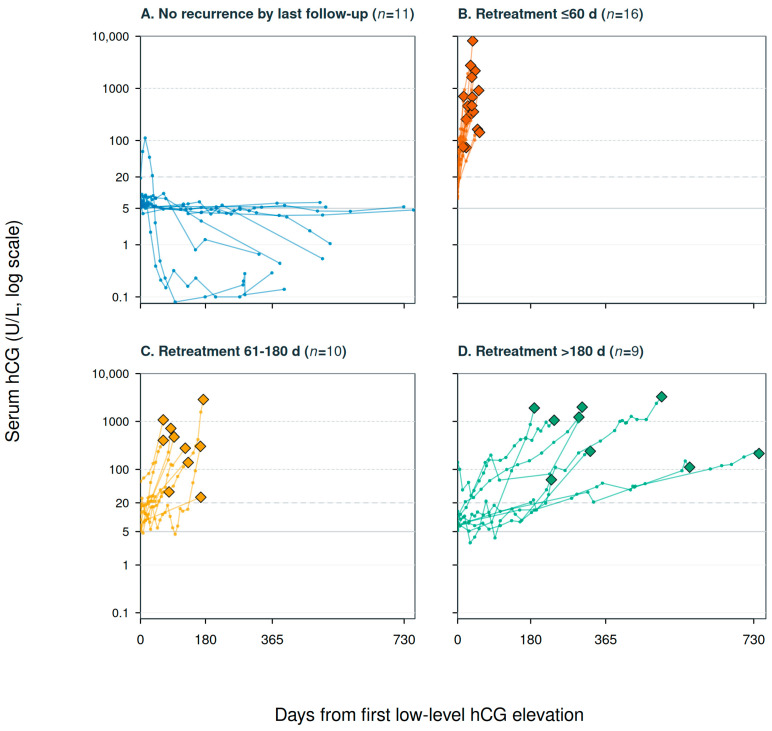
Patient-level hCG trajectories by timing of recurrence and anti-GTN retreatment. Each panel displays one clinical course group, and each thin line represents one patient. The *x*-axis shows days after first low-level hCG elevation, and the *y*-axis shows serum hCG on a log scale (U/L). Diamonds mark initiation of anti-GTN retreatment. Horizontal reference lines are placed at 5, 20, 100, and 1000 U/L. The shared *x*-axis extends to 760 days and includes the latest treatment event at day 743. hCG denotes human chorionic gonadotropin; GTN, gestational trophoblastic neoplasia.

The recurrence group differed from the no-recurrence group in menopausal status, prior GTN diagnosis, FIGO score, and first low-level hCG value (Table 1). Postmenopausal status was recorded in 14.3% versus 45.5% (*p* = 0.043) and choriocarcinoma in 85.7% versus 45.5% (*p* = 0.013). Patients with recurrence had higher FIGO scores (median 9 versus 4; high-risk score in 77.1% versus 27.3%; *p* = 0.004). The median first low-level hCG value was 8.6 U/L (IQR 6.5–19.0), with values of 12.2 U/L (IQR 6.7–33.0) in patients with recurrence and 6.0 U/L (IQR 5.3–6.9) in those without recurrence (*p* = 0.002). All 11 patients whose first low-level hCG value exceeded 20 U/L developed recurrence. Patient-level hCG trajectories are shown in Figure 2.

Among the 35 patients with recurrence, suspicious imaging findings were documented during the recurrence episode in 30 (85.7%). Imaging modalities included CT in 25 patients, MRI in nine, PET/CT in four, and ultrasound in two; modalities could overlap. A numeric maximum lesion diameter was available for 21 of the 30 patients, with a median of 1.76 cm (range 0.70–6.10). Sites of suspicious findings, using the 35 recurrence patients as the denominator and allowing overlap, were lung in 23 patients (65.7%), uterus or pelvis in eight (22.9%), intracranial in four (11.4%), and bone in one (2.9%). All 35 patients who received retreatment subsequently achieved at least one hCG value of 5 U/L or lower.

### 3.2. Treatment-Free Interval and Landmark Analysis Results

The median time from the first low-level hCG elevation to retreatment was 63 days (IQR 36–182). Clinical follow-up from the first low-level hCG elevation among the 35 patients with recurrence had a median duration of 667 days (IQR 331–1256). Among the 11 patients without recurrence by the last available record, the median observation time was 756 days (IQR 505–1304). Five of these 11 patients were postmenopausal. These five patients had hCG values within 3.66–8.48 U/L during 497–1332 days of observation after the first low-level hCG elevation, without interval anti-GTN treatment.

**Figure 3:**
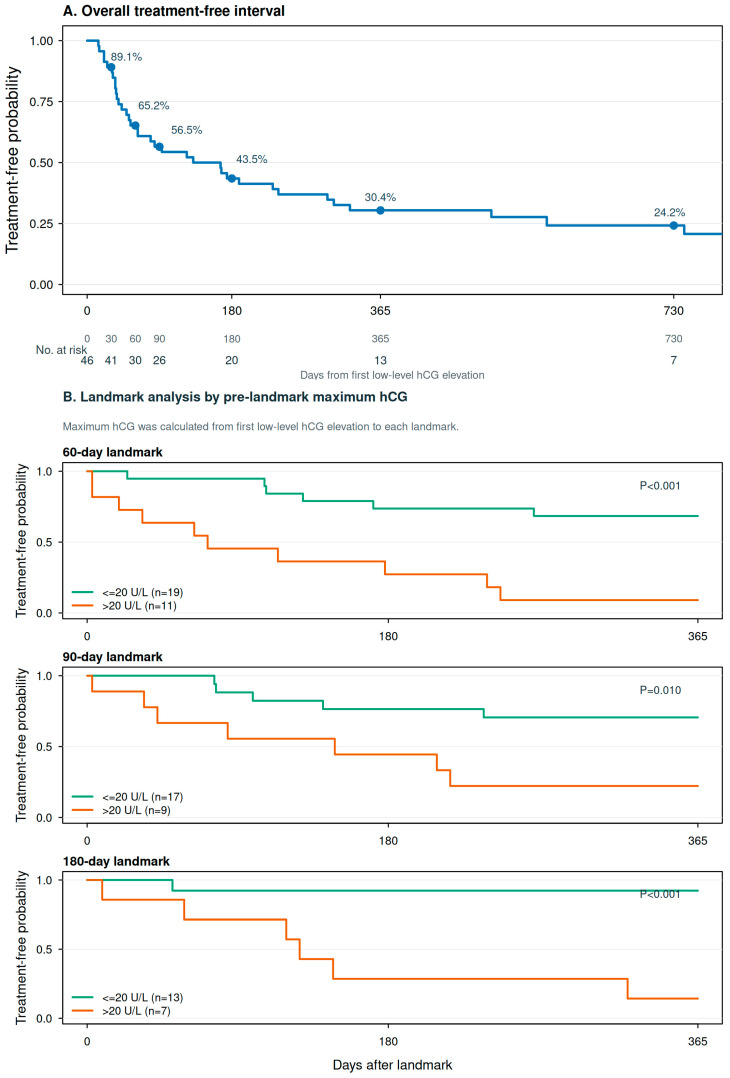
Overall treatment-free probability and landmark analysis stratified by pre-landmark peak hCG. (**A**) shows the Kaplan–Meier estimate from first low-level hCG elevation to retreatment for recurrence. (**B**) shows the landmark analysis using the 60-, 90-, and 180-day landmarks as new time origins and stratifies patients by the highest hCG value recorded from first low-level hCG elevation to the landmark (≤20 versus >20 U/L). *p* values are from log-rank tests censored at 365 days after each landmark. hCG denotes human chorionic gonadotropin.

Using the first low-level hCG elevation as time zero, the treatment-free probability was 89.1% (95% CI 75.8–95.3%) at 30 days, 65.2% (95% CI 49.6–77.0%) at 60 days, 56.5% (95% CI 41.1–69.4%) at 90 days, 43.5% (95% CI 29.0–57.1%) at 180 days, 30.4% (95% CI 18.0–43.9%) at 365 days, and 24.2% (95% CI 12.5–38.0%) at 730 days (Figure 3A).

At the 60-day landmark, 30 patients remained free from retreatment. Nineteen had a pre-landmark peak hCG of 20 U/L or lower, and 11 had a peak above 20 U/L. The 365-day treatment-free probabilities after the landmark were 68.4% (95% CI 42.8–84.4%) and 9.1% (95% CI 0.5–33.3%), respectively (*p* < 0.001).

At the 90-day landmark, 26 patients were included. Seventeen had a pre-landmark peak hCG of 20 U/L or lower and nine had a peak above 20 U/L. The corresponding 365-day treatment-free probabilities were 70.6% (95% CI 43.1–86.6%) and 22.2% (95% CI 3.4–51.3%) (*p* = 0.010).

At the 180-day landmark, 20 patients were included. Thirteen had a pre-landmark peak hCG of 20 U/L or lower and seven had a peak above 20 U/L. The 365-day treatment-free probabilities were 92.3% (95% CI 56.6–98.9%) and 14.3% (95% CI 0.7–46.5%) (*p* < 0.001). The landmark analysis results are summarized in Table 2 and Figure 3B.

### 3.3. Association of Pre-Landmark Peak hCG with Subsequent Events

In the Firth penalized Cox models, a pre-landmark peak hCG >20 U/L was associated with subsequent retreatment at each landmark. At 60 days, the univariable HR was 5.51 (95% CI 2.04–16.05). After separate adjustment for the FIGO score, log10 first low-level hCG, menopausal status, or prior diagnosis, the estimates ranged from 5.27 to 7.05, with all CIs excluding 1. At 90 days, the univariable HR was 3.99 (95% CI 1.31–12.91), and the separately adjusted estimates were in the same direction but less precise. At 180 days, the univariable HR was 12.18 (95% CI 2.51–118.21). Full model results are provided in Supplementary Table S1.

When recurrence events were limited to the 30 patients with suspicious imaging findings, the univariable Firth HR for a pre-landmark peak hCG > 20 U/L was 4.62 (95% CI 1.61–13.92) at 60 days, 3.49 (95% CI 1.09–11.60) at 90 days, and 12.18 (95% CI 2.51–118.21) at 180 days. Kaplan–Meier estimates, log-rank tests, and Firth results are provided in Supplementary Table S2.

## 4. Discussion

### 4.1. Principal Findings

In this cohort of 46 patients with persistent low-level hCG after GTN remission, recurrence requiring retreatment occurred in 35, yet the interval to treatment ranged widely, and 11 patients remained without recurrence over prolonged observation. Treatment-free probability was 30.4% at 1 year and 24.2% at 2 years. Persistent low-level hCG in this setting was therefore usually recurrent disease, but the clinical course was not uniform. At each landmark, patients whose peak hCG had remained at or below 20 U/L had substantially higher treatment-free probability over the following year than those whose hCG had exceeded that level.

### 4.2. Clinically Diagnosed Recurrence and Treatment-Free Time

Recurrence in this cohort was supported by imaging and biochemical response to treatment. Thirty of 35 retreated patients had suspicious imaging findings, most commonly pulmonary lesions. The remaining five were diagnosed clinically from hCG dynamics, prior disease history, and clinical assessment. All 35 achieved hCG ≤ 5 U/L after retreatment. This combination of imaging-supported diagnosis and universal biochemical response is consistent with biologically active trophoblastic disease.

Although most patients eventually received retreatment, the time to treatment varied considerably. In a national cohort of over 4000 patients treated with chemotherapy for GTN, Balachandran et al. showed that most hCG-detected relapses occurred within the first year of surveillance [9]. The present study addresses a later decision point, after remission has been followed by persistent low-level hCG elevation. Some patients entered treatment within weeks, while others were observed for months or longer before the clinical picture warranted intervention. Figure 2 shows the patient-level variation. The landmark analysis then tested whether the early hCG course could distinguish patients requiring early retreatment from those in whom prolonged observation remained feasible.

### 4.3. Early hCG Course and Landmark Analysis

Across all three landmarks, patients whose highest recorded hCG remained at or below 20 U/L had higher treatment-free probability over the subsequent year. The 365-day treatment-free probability ranged from 68.4% to 92.3% in the ≤20 U/L group and from 9.1% to 22.2% in the >20 U/L group. The 20 U/L value was derived empirically from this cohort and served as an exploratory summary marker of the accumulated hCG course from the first low-level elevation to the landmark. When the peak hCG stayed within this range, many patients remained treatment-free for the following year.

Landmark analysis was chosen because the pre-landmark peak hCG is only knowable in patients who have already been observed to that time point without receiving treatment. Assigning this variable at baseline would introduce immortal time bias [4,6]. Fixed landmarks have also been used in GTN hCG kinetics research, including day-50 analyses after methotrexate initiation [5]. In the present study, the landmark approach used a simpler clinical measure, the highest hCG observed before the selected time point among patients still under observation. This mirrors clinical practice, in which the decision to observe or treat is updated as information accumulates. In exploratory Firth models, the association at 60 days remained after the FIGO score, first low-level hCG, menopausal status, or prior diagnosis was added separately. The 90-day estimates were in the same direction but less precise.

Current FIGO and ESGO guidance outlines the evaluation and management of non-tumor causes of persistent low-level hCG [1,2]. In the narrower setting of persistent low-level hCG after GTN remission, the landmark analyses in this study describe treatment-free probability during continued observation. Future studies could assess whether hCG monitoring intervals can be individualized according to hCG level and trajectory.

### 4.4. Non-Recurrent Explanations for Low-Level hCG in This Cohort

In the present cohort, recurrence was the predominant explanation. Serial hCG trajectories, imaging findings, and the previous GTN course were used to distinguish quiescent GTD from early recurrence. Among the retreated patients, suspicious imaging findings were present in 30 of 35, and subsequent hCG normalization in all 35 provided additional support for the diagnosis of recurrence. This clinical pattern placed phantom hCG, heterophilic antibody interference [10,11], and long-term quiescent GTD lower in the differential for these patients. Differences across immunoassay platforms can affect serial hCG interpretation near the positive threshold [12,13,14], but the magnitude of hCG elevation and the imaging findings in most retreated patients make isolated assay discordance unlikely as the sole explanation.

Among the 11 patients without recurrence, five were postmenopausal. These five patients maintained hCG values within a narrow range of 3.66–8.48 U/L over 497–1332 days of observation, without interval anti-GTN treatment. This prolonged narrow low-level pattern is consistent with pituitary hCG production in postmenopausal women [15,16,17]. Pituitary-derived hCG in postmenopausal women typically produces low-level values that overlap with the upper end of the non-pregnant reference range [18,19]. This study did not include gonadotropin suppression testing for these patients, so pituitary origin cannot be confirmed from the available data. The pattern still indicates that menopausal status should be considered when interpreting persistent low-level hCG after GTN remission [20].

Transient benign hCG elevations have been reported following chemotherapy for trophoblastic tumors [21]. A small earlier series described 17 patients with residual low-level hCG at completion of GTN chemotherapy; most became undetectable during follow-up, while one later developed a clear rise and received further treatment [22]. That cohort concerned residual hCG at treatment completion, whereas the present patients had achieved remission before the low-level episode. Studies of quiescent GTD have shown that persistent low-positive hCG in referral populations can remain stable for prolonged periods before either resolving or progressing [23,24,25]. Post-molar surveillance data have documented spontaneous hCG normalization in selected patients with raised but falling hCG [26,27] and risk-adapted follow-up after hCG normalization in complete mole [28]. These reports explain why selected low-hCG patterns may be observed without immediate treatment. The present cohort differs in that all patients had already achieved complete remission after GTN treatment before developing persistent low-level hCG, a later scenario than post-molar surveillance.

### 4.5. Limitations

This study was retrospective and single-center. Persistent low-level hCG elevation after GTN remission is uncommon, and the resulting sample was small, particularly in the no-recurrence group and in the 180-day landmark analysis. Precision was limited at the later landmark, where confidence intervals were wide. The cohort was enriched for high-risk, stage III/IV, drug-resistant, or relapsed disease, consistent with the referral pattern at this center.

Treatment decisions were made in routine clinical practice and reflected physician judgment, patient preference, imaging availability, and the clinical history of complex GTN. The time-to-event endpoint was initiation of retreatment, and hCG contributed to that clinical decision as well as to the pre-landmark exposure. The direction of the results was unchanged when events were limited to the 30 patients with suspicious imaging findings, but incorporation bias remains possible. The cohort size allowed separate adjustment for one prognostic factor at a time, leaving residual confounding by the differences in FIGO score, first low-level hCG, menopausal status, and diagnosis.

Assessment for non-tumor hCG sources followed clinical need and was not based on a uniform protocol. Menopausal status was recorded descriptively and was not systematically assessed with hormonal confirmation. The 20 U/L stratification value was derived post hoc and requires validation in multicenter external cohorts before use in clinical decision-making.

## 5. Conclusions

Most patients with persistent low-level hCG after remission from GTN developed recurrence, although the interval to retreatment varied from weeks to years. Among those still under observation at 60, 90, or 180 days, a peak hCG of 20 U/L or lower before the landmark was associated with higher treatment-free probability over the following year. For these patients, continued surveillance after clinical and imaging assessment is reasonable.

## Figures and Tables

**Table 1:** Baseline and early hCG characteristics by recurrence status.

Characteristic	Overall (*n* = 46)	Recurrence (*n* = 35)	No Recurrence by Last Follow-Up (*n* = 11)	*p* Value
Age at first low-level hCG elevation, years	34 (29–41)	34 (30–40)	41 (31–50)	0.172
Age group				0.065
≤40 years	32 (69.6%)	27 (77.1%)	5 (45.5%)	
>40 years	14 (30.4%)	8 (22.9%)	6 (54.5%)	
Menopausal status				0.043
Postmenopausal	10 (21.7%)	5 (14.3%)	5 (45.5%)	
Premenopausal	36 (78.3%)	30 (85.7%)	6 (54.5%)	
Prior GTN diagnosis				0.013
Choriocarcinoma	35 (76.1%)	30 (85.7%)	5 (45.5%)	
Invasive mole	11 (23.9%)	5 (14.3%)	6 (54.5%)	
FIGO stage				1.000
I	7 (15.2%)	5 (14.3%)	2 (18.2%)	
II	1 (2.2%)	1 (2.9%)	0 (0.0%)	
III	33 (71.7%)	25 (71.4%)	8 (72.7%)	
IV	5 (10.9%)	4 (11.4%)	1 (9.1%)	
FIGO score	8 (6–11)	9 (7–10)	4 (3–8)	0.026
FIGO risk score category				0.004
High-risk (≥7)	30 (65.2%)	27 (77.1%)	3 (27.3%)	
Low-risk (0–6)	16 (34.8%)	8 (22.9%)	8 (72.7%)	
Background of drug-resistant or relapsed GTN				0.702
Yes	33 (71.7%)	26 (74.3%)	7 (63.6%)	
No	13 (28.3%)	9 (25.7%)	4 (36.4%)	
First low-level hCG, U/L	8.6 (6.5–19.0)	12.2 (6.7–33.0)	6.0 (5.3–6.9)	0.002
First low-level hCG category				0.044
≤20 U/L	35 (76.1%)	24 (68.6%)	11 (100.0%)	
>20 U/L	11 (23.9%)	11 (31.4%)	0 (0.0%)	

Note: Values are median (interquartile range) or n (%). Percentages use the column denominator unless otherwise specified. *p* values were calculated using Fisher’s exact test for categorical variables and the Wilcoxon rank-sum test for continuous variables. hCG denotes human chorionic gonadotropin; GTN, gestational trophoblastic neoplasia; FIGO, International Federation of Gynecology and Obstetrics.

**Table 2:** Landmark analysis of 365-day treatment-free probability.

Landmark	Pre-Landmark Peak hCG	Analysis Set, *n*	Anti-GTN Treatment Within 365 Days, *n/N* (%)	365-Day Treatment-Free Probability, KM % (95% CI)	Log-Rank *p*
60 d	≤20 U/L	19	6/19 (31.6%)	68.4 (42.8–84.4)	*p* < 0.001
	>20 U/L	11	10/11 (90.9%)	9.1 (0.5–33.3)	
90 d	≤20 U/L	17	5/17 (29.4%)	70.6 (43.1–86.6)	*p* = 0.010
	>20 U/L	9	7/9 (77.8%)	22.2 (3.4–51.3)	
180 d	≤20 U/L	13	1/13 (7.7%)	92.3 (56.6–98.9)	*p* < 0.001
	>20 U/L	7	6/7 (85.7%)	14.3 (0.7–46.5)	

Note: Each analysis included only patients who reached the respective landmark without prior anti-GTN treatment for recurrence. The landmark was used as the new time zero. Blank cells in the landmark and log-rank P columns refer to the same landmark comparison as the row above. Event counts represent the observed numbers of events; KM estimates are censoring-adjusted. hCG denotes human chorionic gonadotropin; KM, Kaplan–Meier; CI, confidence interval.

## Data Availability

De-identified aggregate data used in this study may be made available by the corresponding author upon reasonable request and with approval from Peking Union Medical College Hospital. Individual patient-level data are not publicly available due to privacy and ethical restrictions.

## Supplementary Materials

The following supporting information can be downloaded at: https://www.mdpi.com/article/10.3390/cancers18152434/s1, Table S1. Association of pre-landmark peak hCG >20 U/L with subsequent anti-GTN retreatment in Firth penalized Cox models. Table S2. Landmark results with imaging-supported recurrence as the event.
