# Supplementary material for: Persistent Low-Level hCG After Gestational Trophoblastic Neoplasia Remission: Treatment-Free Probability Stratified by Early hCG Course
Source: Cancers (Basel). 2026 Jul 29;18(15):2434. doi: 10.3390/cancers18152434 (PMC13464989; doi:10.3390/cancers18152434)
Supplement: Supplementary file 1 [file cancers-18-02434-s001.zip › cancers-4409996-supplementary.pdf]

Supplementary Materials

Persistent Low-Level hCG After Gestational Trophoblastic Neoplasia Remission: Treatment-Free Probability Stratified by Early hCG Course

Supplementary Table S1. Association of pre-landmark peak hCG >20 U/L with subsequent anti-GTN retreatment in Firth penalized Cox models.

Land-mark	Analysis set, n	Events within 365 days, n	Model	HR (95% CI)	P value
60 days	30	16	Univariable	5.51 (2.04–16.05)	<0.001
			Adjusted separately for FIGO score	5.27 (1.94–15.57)	0.001
			Adjusted separately for log10(first low-level hCG)	7.05 (2.11–24.52)	0.002
			Adjusted separately for menopausal status	5.29 (1.83–17.69)	0.002
			Adjusted separately for diagnosis	5.76 (1.95–18.93)	0.001
90 days	26	12	Univariable	3.99 (1.31–12.91)	0.015
			Adjusted separately for FIGO score	3.57 (1.14–11.91)	0.029
			Adjusted separately for log10(first low-level hCG)	4.49 (1.07–18.63)	0.040
			Adjusted separately for menopausal status	2.63 (0.87–8.50)	0.086
			Adjusted separately for diagnosis	4.00 (1.18–15.53)	0.026
180 days	20	7	Univariable	12.18 (2.51–118.21)	0.001

Note: The hazard ratio compares pre-landmark peak hCG >20 U/L with ≤20 U/L. All models used 365-day post-landmark follow-up. Adjusted models added one covariate at a time. First low-level hCG was log10-transformed and interpreted per 10-fold increase. Confidence intervals are profile-likelihood 95% CIs; P values are likelihood-ratio P values. At the 90-day landmark, all five postmenopausal patients were in the ≤20 U/L group and had no events. hCG denotes human chorionic gonadotropin; FIGO, International Federation of Gynecology and Obstetrics; HR, hazard ratio; CI, confidence interval.

Supplementary Table S2. Landmark results with imaging-supported recurrence as the event.

Landmark	Pre-landmark peak hCG	Analysis set, n	Imaging-supported recurrence within 365 days, n/N (%)	365-day event-free probability, KM % (95% CI)	Log-rank P	Firth HR (95% CI); P value
60 days	≤20 U/L	19	6/19 (31.6%)	68.4 (42.8–84.4)	0.002	Reference
	>20 U/L	11	8/11 (72.7%)	12.6 (0.7–42.1)		4.62 (1.61–13.92); 0.005
90 days	≤20 U/L	17	5/17 (29.4%)	70.6 (43.1–86.6)	0.028	Reference
	>20 U/L	9	6/9 (66.7%)	26.7 (4.1–57.9)		3.49 (1.09–11.60); 0.036
180 days	≤20 U/L	13	1/13 (7.7%)	92.3 (56.6–98.9)	<0.001	Reference
	>20 U/L	7	6/7 (85.7%)	14.3 (0.7–46.5)		12.18 (2.51–118.21); 0.001

Note: An event was counted only when suspicious imaging findings were documented during the recurrence episode; the original treatment-decision date was retained as the event date. Each analysis included patients who reached the landmark without an earlier event. Event counts represent the observed numbers of events; Kaplan-Meier estimates account for censoring. The Firth hazard ratio compares pre-landmark peak hCG >20 U/L with ≤20 U/L. Confidence intervals are profile-likelihood 95% CIs; Firth P values are likelihood-ratio P values. hCG denotes human chorionic gonadotropin; KM, Kaplan-Meier; HR, hazard ratio; CI, confidence interval.
